# Pain Intensity and Degree of Disability after Fragility Fractures of the Pelvis

**DOI:** 10.3390/medicina58040477

**Published:** 2022-03-25

**Authors:** Alexandru Filip, Bogdan Veliceasa, Bogdan Puha, Nina Filip, Elena Cojocaru, Mihaela Pertea, Claudiu Adrian Carp, Bogdan Huzum, Ovidiu Alexa, Pol Maria Rommens

**Affiliations:** 1 Department of Orthopedics and Traumatology, Surgical Science (II), Faculty of Medicine, “Grigore T. Popa” University of Medicine and Pharmacy, 700020 Iasi, Romania; alexandru.filip@ymail.com (A.F.); puhab@yahoo.com (B.P.); adrianclaudiucarp@gmail.com (C.A.C.); bogdan.huzum93@gmail.com (B.H.); ovidiu.alexa@umfiasi.ro (O.A.); 2 Department of Morpho-Functional Sciences (II), Faculty of Medicine, “Grigore T. Popa” University of Medicine and Pharmacy, 700020 Iasi, Romania; 3 Department of Morpho-Functional Sciences (I), Faculty of Medicine, “Grigore T. Popa” University of Medicine and Pharmacy, 700020 Iasi, Romania; ellacojocaru@yahoo.com; 4 Department Plastic Surgery and Reconstructive, Surgical Sciences (I), Faculty of Medicine, Grigore T. Popa University of Medicine and Pharmacy, 700020 Iasi, Romania; pertea_mihaela@yahoo.com; 5 Department of Orthopedics and Traumatology, University Medical Center of Johannes Gutenberg-University Mainz, 55131 Mainz, Germany; pol.rommens@unimedizin-mainz.de

**Keywords:** osteoporosis, mobility, pelvic fractures, daily activities

## Abstract

*Background and objectives*: Fragility fractures of the pelvis (FFP) are of increasing interest lately, being associated with a loss of mobility and affecting the quality of life. The aim of our study was to investigate the effect of FFP on disability and pain in patients, after one year since injury. *Materials and Methods*: In the study, we included 76 patients diagnosed with FFP, who were admitted to our trauma department between January 2016 and January 2019, and were above 65 years of age. The Von Korff pain intensity and disability scores were calculated in the hospital at 6 months and after 1 year. *Results*: Fifty-four patients were female (71%), with an average age of 75.9 ± 7.19 years. Twenty-two patients were male (29%) and had a mean age of 77.22 ± 7.33 years. We did not record significant differences regarding age between the men and women (*p* > 0.05). Significant improvements appeared between the baseline and the 6 month follow-up; the average pain intensity score at 6 months was 44.94 (SD 21.20) (*p* < 0.001), and the disability score was 54.30 (SD 21.62). The following average pain intensity and disability scores after 12 months were similar to the values at6 months: 44.48 (SD 21.74) for pain intensity and 52.36 (SD 24.53) for disability. The Von Korff pain score at 6 months and after 1 year depends on gender and on the initial Von Korff pain score (*p* = 0.02). The Von Korff disability score at 6 months depends on gender, the baseline pain score and the baseline disability score (*p* = 0.001). *Conclusions*: our patients reported long-lasting pain that had a severe effect on their daily routines, and they could not return to their normal status prior to injury.

## 1. Introduction

Because of the high life expectancy in our population, fragility fractures have become more frequent [1]. Bone fragility is a severe medical condition linked to osteoporosis, loss of mobility and patient independence, and is associated with increased mortality [2].

Fragility fractures are defined as fractures that occur following a low-energy trauma [3]. The fracture occurs as a consequence of an alteration to the bone structure, which leads to a loss of the mechanical properties (resistance to compression, torsion, and elasticity) of the bone. Women over the age of 60 have the highest frequency of fragility fractures [4].

The priority in the treatment of fragility fractures of the pelvis (FFP) is pain control and the restoration of mobility. Bone strength and bone morphology are restored by means of conservative and surgical management. Pharmacotherapy is involved in bone resorption through anti-resorptive and anabolic medication. Agents such as calcium, bisphosphonates, denosumab, selective estrogen receptor modulators and estrogen hormone therapy (raloxifene) inhibit bone resorption, and agents such as teriparatide and abaloparatide promote bone mass formation.

A third anabolic product (romosozumab) is in the FDA approval procedure [5,6]. Osteonecrosis of the jaw, atypical femur fractures in the case of biphosphonate administration, renal calculi in hypercalcemia and thrombotic events in the case of hormone therapy are some of the risks due to the side effects of antiosteoporotic drugs [7,8,9].

Pain and disability are the primary problems caused by FFP in elderly people. Many studies indicate long periods of immobility and, therefore, a lack of independence for patients who suffer from FFP [10,11,12]. The choice between surgical and conservative treatment is given by the type of fracture and the patient’s comorbidities [13,14,15,16].

In recent years, there has been an increased frequency of fragility fractures in the elderly. These events are associated with prolonged hospitalizations, disability, morbidity and last, but not least, mortality. In this context, the aim of our study was to investigate the effect of FFP on disability and pain in patients, after one year since injury.

## 2. Materials and Methods

In our observational study, we enrolled patients with an age higher than 65 years, who were admitted to our hospital with FFP, from January 2016 to January 2019. Only the patients who were treated conservatively were recruited. The fractures were diagnosed by X-ray or CT scan. Exclusion criteria for the patients included treatment with anti-osteoporotic drugs or prior pelvic fractures. We also ruled out pelvic fractures due to high-energy mechanisms or metastatic pathology. We informed all our patients about information regarding the study and provided invitations for participation. For those who agreed to take part in the study, questionnaires were given to them during hospital admission. Upon admission, each patient was given an anticoagulant and analgesic regimen. The analgesic therapy and cocktail were adapted according to the pathology of each patient and to their tolerance to these drugs. The analgesic regimen consisted of Urgendol, Quamatel, Ketoprofen and Perfalgan, all in a two-vial dosage. The evaluations at 6 and 12 months were conducted by interviewing patients by phone. Informed consent was obtained from all patients who accepted to participate in our study. The research was carried out in conformity with the Declaration of Helsinki and authorized by the Ethical Board of the Emergency Hospital “Sf. Spiridon” in Iasi (8 October 2019).

### 2.1. Questionnaire

#### Von Korff Pain Intensity and Disability Questionnaire

Von Korff et al. [17] developed a simple and short questionnaire to assess the severity of chronic pain problems, known as the Chronic Pain Grade (CPG). The questionnaire is self-administered and validated for use in patients with pain outside of hospital [17,18]. The questionnaire used in this study consists of six questions; three questions evaluate the intensity of the current pain, the average pain and the most severe pain, and three questions evaluate disability items. The pain intensity score is evaluated as the average of the three 0–10 ratings and is multiplied by 10 to yield a 0–100 score. Decreased pain is represented by low scores. The pain intensity score is calculated in a similar manner to the disability score [17,18,19]. The Von Korff pain intensity and disability questionnaire is provided in the Supplementary Material.

### 2.2. Statistical Analysis

We used SPSS version 18 for the statistical analysis. Data were presented as mean ± standard (SD) deviation or percentage (±SD). Statistical significance was defined as a *p* < 0.05.

The multiple correlation coefficient, as a measure of the association between y and the set of variables x, introduces the multiple correlation coefficient, denoted by R. It can be defined as the maximum simple correlation coefficient (Pearson) between y and a linear combination of variables x. This explains why the calculated value of R is always positive and tends to increase as the number of independent variables increases. The least squares method can, thus, be thought of as a method that maximizes the correlation between the observed values and the estimated values (those representing a linear combination of variables x). An R value close to 0 denotes an insignificant regression, meaning that the predicted regression values are not any better than those obtained by a random approximation (or based only on the distribution of y).

## 3. Results

From January 2016 to January 2019, we admitted 112 patients with FFP to our hospital. A total of 29 patients refused to participate and were excluded. Ultimately, we included 83 patients in the study. Only 76 patients responded to the questionnaires at all three evaluations; 5 did not respond to the questionnaires at all and 2 died during the follow-up period.

The mean age of the patients was 76.28 ± 7.26 years. We did not record significant differences regarding age between the men and women (*p* > 0.05). 

The general characteristics for all the patients are presented in Table 1.

**Figure 1:**
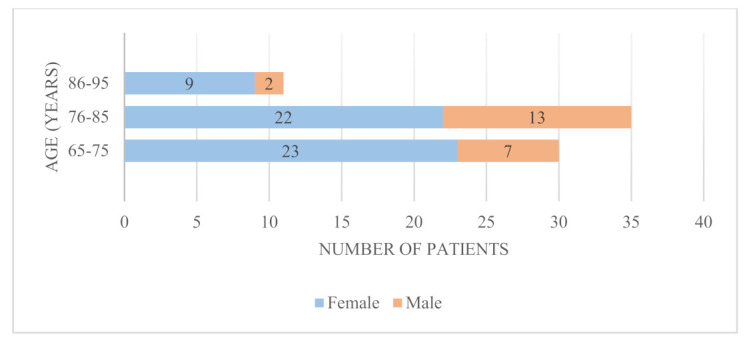
Sex and age distribution of patients included in the study.

The age distribution of the patients with FFP enrolled in our study, by gender, is presented in Figure 1.

### Fracture Type

**Figure 2:**
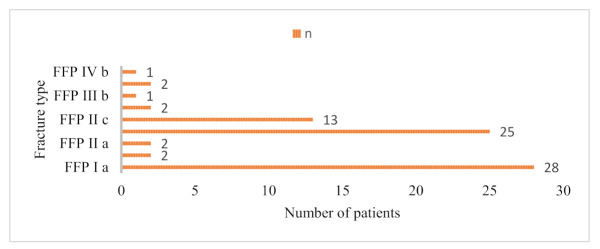
Distribution and numbers of FFP type.

Following the Rommens and Hofmann classification of the FFP, 28 patients had FFP type Ia lesions (unilateral anterior lesions), 2 had FFP type Ib lesions (bilateral anterior lesions), 2 had FFP type IIa lesions (non-displaced sacral crush, without anterior disruption), 25 had FFP type IIb lesions (non-displaced sacral crush, with anterior disruption), 13 had FFP type IIc lesions (non-displaced sacral, sacroiliac or iliac fracture, with anterior disruption), 2 had type IIIa lesions (displaced unilateral iliac fracture), 1 had a type IIIb lesion (displaced unilateral iliosacral disruption), 2 had type IIIc lesions (displaced unilateral sacral fracture), and 1 had a type IVb lesion (bilateral sacral fracture, with spinopelvic dissociation) [10]. The distribution and numbers of FFP type in the patients included in our study (*n* = 76) are presented in Figure 2.

The results of the questionnaires at all three evaluation times are presented in Table 2.

The data are given as mean ± SD, and the P values represent the differences between 6 months, 1 year and baseline.

**Figure 3:**
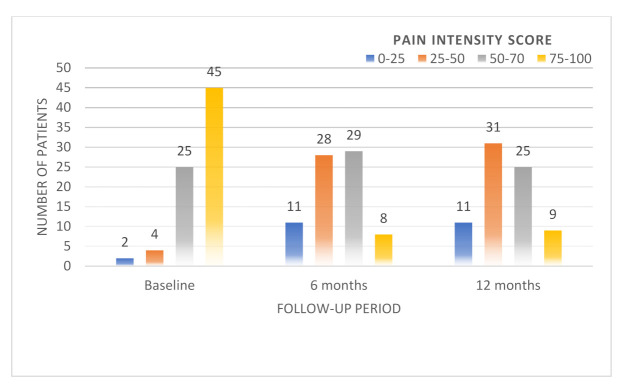
Distribution of Von Korff pain scores during the first year after injury.

**Figure 4:**
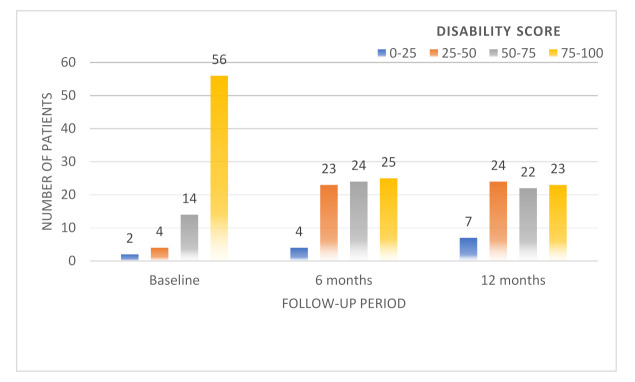
The distribution of Von Korff disability scores over one year.

The distribution of Von Korff pain and disability scores during the first year after injury can be observed in Figure 3 and Figure 4.

The initial pain intensity score was found to be between 75 and100 in 45 patients (59.2%), between 50 and75 in 25 patients (32.9%), and between 25 and50 in 4 patients (5.3%). Only 2 (2.6%) patients had a score of less than 25 points. Pain severity decreased considerably at the 6-month follow-up, from 59.2% to 10.5% (*p* < 0.001), and remained elevated at the 1-year follow-up, at 11.8%.

A disability score higher than 75 was reported by 73.7% of the patients in the hospital, 32.9% of the patients at the 6-month follow-up, and by 30.3% of the patients at the 1-year follow-up.

The multivariate analysis demonstrates the correlation between sex, initial pain, and disability scores during the evolution of FFP at 6 months and at 12 months (Table 3).

A percentage of 74.2% of the variation in pain scores at 6 months is explained by the variables gender and baseline Von Korff pain scores (*p* = 0.001).

Only 29.4% of the variation in disability scores at 6 months is correlated with the variables gender and baseline Von Korff pain and disability score, but the result was not statistically significant (*p* = 0.087).

The results of the multiple linear regression analysis, which considers gender and the correlation between baseline pain score and disability score, are presented in Table 4.

The specific analyses showed that the Von Korff pain score at 6 months and after 1 year depends on sex and on the initial Von Korff pain score (*p* = 0.02). The disability score at 6 months is dependent on sex (y = 88.09 − 25,923 gender − 0.251 baseline pain score +0.391 baseline disability score, *p* = 0.001). The disability score at 12 months apparently does not depend significantly on sex, the baseline pain score or the baseline disability score (*p* = 0.662).

## 4. Discussion

Upon admission, the patients suffered from severe immobilizing pain. In a previous study, in which we evaluated the correlation between pain and early mobilization, most patients reported a decrease in pain intensity after 14 days [20]. An indication of favorable development is the remission of pain within the first weeks. Less pain allows for early and progressive mobilization. In the case of FFP, mobilization with limited and progressive weight bearing is recommended. Simultaneously, the pain level is assessed and X-ray controls are performed [21].

In our study, the incidence of FFP in females (71%) was significantly higher than in males (29%). In a retrospective analysis of 245 patients with FFP, a similar distribution was found when patients were stratified by sex [21].

Most of the studies referring to the follow-up of patients who suffered from FFP were focused on the functional outcome and mortality rate [22,23,24,25,26,27]. The loss of independence and immobilization described pain as an indirect cause. The study published by Hoch et al. [28] compared the outcome and mortality rate of non-operative and operative treatment of FFP. The obtained results indicated the same pain level (using the visual analogue scale) at the 2-year follow-up for both groups [28].

The results of our study show an important decrease in the pain intensity score (44.94 ± 21.20) at 6 months, compared to baseline (73.19 ± 18.21, *p* < 0.001). At the one-year follow-up, the pain intensity score (44.48 ± 21.74) was low compared to baseline (73.19 ± 18.21, *p* < 0.001), but had not significantly changed from the 6-month follow-up. The pain levels after 6 months and after 12 months were very similar. This indicates that the pain intensity diminished earlier. In further studies, we suggest that the researchers evaluate the pain intensity at earlier times (6 weeks and 3 months, or 4, 8 and 12 weeks) to have a better idea of the natural course of pain intensity after FFP.

Similar results were also obtained when assessing the Von Korff disability score. Suzuki et al. reported similar results when they evaluated the course of pain intensity and degree of disability in the first year after acute vertebral compression fractures were treated non-surgically [19].

The participants who underwent our questionnaires and were involved in the study, and who described an active lifestyle prior to their fragility fracture, reported being unable to achieve similar resistance levels and intensity regarding physical activities.

Although the intensity of the pain decreased compared to baseline, some pain remained, which had a negative impact on their quality of life.

A study about the experience of women who suffered from post-menopausal vertebral fractures reported that pain from their fracture severely restricted their routine social activities [29]. The pain intensity did not return to zero or to a very low level. This means that patients with FFP and conservative treatment suffer from permanent pain. The same can be said for disability; there is a permanent degree of disability. An interesting question would be the following: “Is this also the case after operative treatment?”.

The development of rehabilitation strategies that alleviate pain and improve the quality of life for patients suffering from FFP is a major necessity. New rehabilitation strategies that alleviate pain and improve the quality of life require a combination of therapeutic exercise with continuous radiofrequency or the administration of food supplements [30,31,32].

This study has limitations. The patients were not clinically consulted to assess the outcomes. 

Our study highlights the importance of the diagnosis and treatment of osteoporosis to reduce and prevent the risk of FFP. To reduce healthcare-related costs in the future, it is important to focus on the prevention of falls and osteoporosis.

The questionnaire of Von Korff is very simple. A more detailed questionnaire could provide the researcher and reader with a more differentiated view of what kind of disability is permanent. The therapists could develop specific treatment protocols with this new knowledge.

## 5. Conclusions

The patients included in our study reported long-lasting pelvic pain, which they described as disturbing, and they reported that this pain affected their daily activities- and that they were ultimately unable to achieve the same quality of life. It is clear from our results that FFP is directly related to the loss of social and physical independence. Efforts are needed to prevent pelvic insufficiency fractures. The development of rehabilitation strategies that alleviate pain and improve the quality of life for patients suffering from FFP is a necessity. 

## Figures and Tables

**Table 1:** Characteristics of the patients.

Characteristic	Patients (*n* = 76)
Age (years)	76.28 ± 7.26 (66–92)
Male age (years)	77.22 ± 7.33
Female age (years)	75.9 ± 7.19
Height (cm)	169 ± 11.70
Weight (kg)	81 ± 9.85
Current smokers (%)	31.21
Coffee consumption (˃3 cups/d, %)	43.51
Domestic fall (%)	67.10
Unknown trauma (%)	3.94
No history of trauma (%)	28.94
Days in hospital	7.53 ± 2.60

**Table 2:** Von Korff scores at baseline, at 6 months and after 1 year.

	Baseline	6 Months	One Year
Von Korff pain score	73.19 ± 18.21	44.94 ± 21.20 (*p* < 0.001)	44.48 ± 21.74 (*p* < 0.001)
Von Korff disability score	74.14 ± 15.18	54.30 ± 21.62 (*p* < 0.001)	52.36 ± 24.53 (*p* < 0.001)

**Table 3:** Multivariate analysis between gender, initial Von Korff pain and disability scores, and in the Von Korff pain and disability scores at 6 months and at 12 months.

Model	R	R Square	Adjusted R Square	Std. Error of the Estimate	Change Statistics				
					R Square Change	F Change	df1	df2	Sig. F Change
**DEPENDENT VARIABLE: PAIN SCORE 6 MONTHS**									
**1**	0.703 (a)	0.494	0.487	15.188	0.494	72.178	1	74	0.001
**2**	0.865 (b)	0.749	0.742	10.774	0.255	74.042	1	73	0.001
**3**	0.871 (c)	0.759	0.748	10.634	0.010	2.942	1	72	0.091
**DEPENDENT VARIABLE: PAIN SCORE 12 MONTHS**									
**1**	0.710 (a)	0.504	0.497	15.420	0.504	75.091	1	74	0.001
**2**	0.852 (b)	0.725	0.718	11.552	0.222	58.853	1	73	0.001
**3**	0.859 (c)	0.737	0.726	11.370	0.012	3.346	1	72	0.072
**DEPENDENT VARIABLE: DISABILITY SCORE 6 MONTHS**									
**1**	0.542 (a)	0.294	0.284	18.298	0.294	30.778	1	74	0.001
**2**	0.542 (b)	0.294	0.274	18.422	0.000	0.007	1	73	0.934
**3**	0.568 (c)	0.322	0.294	18.173	0.028	3.012	1	72	0.087
**DEPENDENT VARIABLE: DISABILITY SCORE 12 MONTHS**									
**1**	0.632 (a)	0.399	0.391	19.141	0.399	49.215	1	74	0.001
**2**	0.902 (b)	0.813	0.808	10.758	0.413	161.253	1	73	0.001
**3**	0.902 (c)	0.813	0.805	10.832	0.000	0.000	1	72	0.992

a Predictors: (Constant), Gender; b Predictors: (Constant), Gender, Baseline von Korff pain score; c Predictors: (Constant), Gender, Baseline von Korff pain score, Baseline von Korff disability score.

**Table 4:** Multiple linear regression analysis that considers gender, and the correlation between initial pain score and disability score.

Model		Unstandardized Coefficients		Standardized Coefficients	t	Sig.	95% Confidence Interval for B	
		B	Std. Error	Beta			Lower Bound	Upper Bound
1	Dependent Variable: pain score 6 months							
	(Constant)	23.684	9.989		2.371	0.020	3.771	43.598
	Gender	−19.720	3.090	−0.425	−6.382	0.001	−25.879	−13.560
	Baseline Pain Score	0.523	0.118	0.449	4.446	0.001	0.288	0.757
	Baseline Disability Score	0.226	0.132	0.162	1.715	0.091	−0.037	0.488
2	Dependent Variable: pain score one year							
	(Constant)	27.657	10.681		2.589	0.012	6.365	48.950
	Gender	−21.499	3.304	−0.451	−6.508	0.001	−28.085	−14.913
	Baseline Pain Score	0.471	0.126	0.395	3.751	0.001	0.221	0.722
	Baseline Disability Score	0.258	0.141	0.180	1.829	0.072	−0.023	0.538
3	Dependent Variable: disability score 6 months							
	(Constant)	88.090	17.071		5.160	0.001	54.059	122.121
	Gender	−25.923	5.280	−0.547	−4.910	0.001	−36.449	−15.397
	Baseline Pain Score	−0.251	0.201	−0.212	−1.251	0.215	−0.652	0.149
	Baseline Disability Score	0.391	0.225	0.274	1.736	0.087	−0.058	0.839
4	Dependent Variable: disability score one year							
	(Constant)	4.463	10.176		0.439	0.662	−15.822	24.748
	Gender	−14.535	3.147	−0.270	−4.618	0.001	−20.809	−8.260
	Baseline Pain Score	0.993	0.120	0.737	8.291	0.001	0.754	1.231
	Baseline Disability Score	0.001	0.134	0.001	0.010	0.992	−0.266	0.269

## Supplementary Materials

The Von Korff questionnaire can be downloaded at: https://www.mdpi.com/article/10.3390/medicina58040477/s1.
