# Supplementary material for: Pain Intensity and Degree of Disability after Fragility Fractures of the Pelvis
Source: Medicina (Kaunas). 2022 Mar 25;58(4):477. doi: 10.3390/medicina58040477 (PMC9024514; doi:10.3390/medicina58040477)
Supplement: Supplementary file 1 [file medicina-58-00477-s001.zip › medicina-1647831-supplementary.pdf]

medicina

Von Korff Pain Intensity and Disability Score

Questions Used to Grade Chronic Pain Status and disability

(1) How would you rate your pain on a 0–10 scale at the present time (right now)? [Pain Right Now]

No pain — Pain as bad as it could be

0 1 2 3 4 5 6 7 8 9 10

(2) After the fracture, how intense was your worst pain? [Worst Pain]

No pain — Pain as bad as it could be

0 1 2 3 4 5 6 7 8 9 10

(3) After the fracture, on average, how intense was your pain? (That is, your usual pain at times you were experiencing pain) [Average Pain]

No pain — Pain as bad as it could be

0 1 2 3 4 5 6 7 8 9 10

(4) After the fracture, how much has the pain interfered with your daily activities? [Daily Activities]

No interference — Unable to carry on any activities

0 1 2 3 4 5 6 7 8 9 10

(5) After the fracture, how much has the pain changed your ability to take part in recreational social and family activities? [Social Activities]

No change — Extreme change

0 1 2 3 4 5 6 7 8 9 10

(6) After the fracture, how much has the pain changed your ability to work (including housework)? [Work Activities]

No change — Extreme change

0 1 2 3 4 5 6 7 8 9 10

Pain intensity score = (((response question 1) + (response question 2) + (response question 3))/3)*10

Disability score = (((response question 4) + (response question 5) + (response question 6))/3)*10
